# Effects on meat quality and black bone incidence of elevated dietary vitamin levels in broiler diets challenged with aflatoxin

**DOI:** 10.1017/S1751731119001216

**Published:** 2019-06-03

**Authors:** M. M. Mota, R. G. Hermes, C. S. S. Araújo, A. S. C. Pereira, N. B. P. Ultimi, B. G. S. Leite, L. F. Araújo

**Affiliations:** 1 Department of Animal Science, University of Sao Paulo, Av. Duque de Caxias Norte, 225, Pirassununga, 13634503, Brazil; 2 DSM Nutritional Products, 05321010, Sao Paulo, Brazil; 3 Department of Animal Nutrition and Production, University of São Paulo, Pirassununga, Brazil

**Keywords:** binder, carcass yield, malonaldehyde, mycotoxin, performance

## Abstract

Vitamins play an essential role in broiler nutrition. They are fundamental for normal metabolic and physiological process, and their requirements for poultry are not fixed and can be affected by multiple factors. In contrast, mycotoxins are a challenging issue because they hinder performance and the immune system. Vitamin supplementation above minimum requirements would permit improvement in productive potential, health, bone and meat quality in a situation of mycotoxin challenge. The objective of this study was to determine the influence of optimum vitamin nutrition in diets contaminated with aflatoxin in broilers from 1 to 44 days of age. A total of 1800 Cobb 500 male chicks were randomized to 15 sets of eight treatment groups, each containing 15 birds using a 2 × 2 × 2 factorial design (commercial vitamin levels and high vitamin levels, two levels of aflatoxin – 0 and 0.5 ppm with binder levels of 0 and 10 000 mg/kg). The mash diets were corn and soybean meal based, formulated according to commercial practices. Feed intake, weight gain and feed conversion were analyzed for birds from 1 to 44 days of age. To determine carcass characteristics (carcass yield, breast yield and leg yield) and black bone syndrome, two birds were slaughtered from each group at 45 days. Other analyses included breast tenderness, water loss by dripping and malonaldehyde concentrations. The results demonstrated that broilers that were fed high levels of vitamins showed better weight gain, feed conversion, carcass yield and breast yield than broilers that were fed diets with commercial vitamin levels (*P* < 0.05); also, broilers that were fed diets containing 0.5 ppm aflatoxin had lower weight gain, carcass yield and breast yield (*P* < 0.05). The use of 10 000 mg/kg of binder improved (*P* < 0.05) feed conversion throughout the rearing period. We conclude that aflatoxin negatively affects performance and carcass yield; however, feeding optimum vitamin nutrition improved these performance traits.

## Implications

Dietary vitamin supplementation above the established minimum requirements improves health, performance, bone quality and the welfare of poultry. Optimizing vitamin supplies in broilers minimizes poor bone quality and problems relating to feed contamination with mycotoxin.

## Introduction

In recent years, the poultry sector has undergone significant transformations, resulting in better production indexes and quality of products purchased by consumers. Improved nutrition has been one of the factors driving these transformations. There is a large amount of data showing the nutritional requirements for proteins, amino acids and minerals, such as calcium and phosphorus. However, few studies have evaluated broiler vitamin requirements.

Vitamins are essential nutrients, involved in over 30 metabolic reactions at the cellular level (Marks, 1975). Among other functions, vitamins are involved in metabolism and act as immunomodulators, improving resistance to infections. In general, nutritionists balance rations based on the minimal necessary levels for performance and profit and add a safety margin based on experience. It is worth noting that, in commercial establishments, vitamin supplementation takes into account the production goals, which must be reached at the lowest possible cost, and a safety margin that allows for potential stress factors. In addition to signs of deficiency and performance, new parameters are being analyzed to determine broiler vitamin requirements. These include immune response, well-being, increased carcass vitamin content to improve presentation, shelf life and the nutritional value of meat to consumers.

Higher levels of vitamins in broiler diets compensate for changes in intake, bioavailability, issues that compromise the quality of the food and the level of stress. In general, a significant immune response occurs only when vitamins are supplemented at a level 10 times higher than those recommended by the NRC (1994), or two to three times higher than those commercially used (Leeson, 2007).

According to Felix *et al*. (2009), broiler performance can be improved by higher vitamin levels than those recommended by research centers. However, there is little data and new research is needed to determine the requirements of modern strains of poultry. Since there are many benefits associated with feeding effective levels of vitamins to broilers, the concept of supplying vitamin levels above industry recommendations and nutrition tables has been gaining momentum (Soto-Salanova *et al*., 2010; Mejia *et al*., 2014).

One challenging issue faced by the poultry sector is the use of feed ingredients containing aflatoxin. Mycotoxins hinder poultry performance, compromising the immune system and, in extreme cases, causing death. Aflatoxins are absorbed from the gastrointestinal tract, and within 24 h of ingestion of contaminated feed, concentrations of this toxin in the liver, reproductive organs and kidneys are high (Sawhney *et al*., 1973). Liver damage hampers the metabolism of proteins, carbohydrates and lipids. The use of vitamin levels above those recommended by the NRC can protect against the slowing of animal growth caused by aflatoxins (Santúrio, 2000).

There are a number of commercially available binders that can decrease the effects of grains contaminated with mycotoxins. Binders can physically adhere to aflatoxins and prevent them from being absorbed by the gastrointestinal tract, thus reducing the toxicity to animals (Olver, 1997).

In contrast, black bone syndrome (**BBS**) is a condition that seems to be related to redness of the tissue adjacent to the bone, which can blacken during cooking or storage (Baldo *et al*., 2013). This results in an unappetizing appearance to the meat. For this reason, fast-food suppliers avoid frozen broiler leg portions or use deboned meat instead (Whitehead, 2010). Since vitamin D plays an important role in calcium and phosphorus absorption and, therefore, influences bone quality, the use of higher vitamin levels may help to minimize BBS.

The aim of this study was to analyze the levels of optimum vitamin nutrition (**OVN**) in broiler diets from 1 to 44 days of age, with or without aflatoxin, and their impact on performance, carcass yield, meat quality traits and BBS incidence.

## Materials and methods

The trial took place in the experimental poultry facility at the Faculty of Animal Science and Food Engineering of the University of Sao Paulo, Pirassununga campus (Brazil).

### Animals, experimental design and diets

A total of 1800 one-day-old male Cobb 500 chicks were randomly allocated to 15 sets of eight different treatment groups of 15 birds, using a factorial 2 × 2 × 2 design (levels of vitamin supplementation: control and OVN; levels of aflatoxins: 0 and 0.5 ppm; levels of binder: 0 and 10 000 mg/kg).

The birds were housed in a temperature-controlled 45 × 10 m barn, with negative pressure ventilation and cool-cells with insulated asbestos roofing. There were a total of 120 pens, measuring 1.0 × 1.2 m, and each pen was provided with nipple drinkers, one tube feeder and rice husk litter.

Mashed feed and water were provided *ad libitum* during the whole trial. The barn was heated using an automatic gas heating system, which was activated depending on the internal temperature. The initial temperature was 32°C, and it was gradually decreased according to the housing recommendations for the strain. The lighting system was set according to the Cobb broiler management guide.

The diets were provided in four stages: pre-starter (1 to 7 days), starter (8 to 21 days), grower (22 to 38 days) and finisher (39 to 45 days), formulated according to commercial practices (Table 1). The levels of vitamins used (control and OVN) can be found in Table 2. Control vitamin supplementation considered the current average industry levels in Sao Paulo, Brazil. Deactivated bentonite and yeast-based binder was used in the pre-starter and starter diets only. Aflatoxin used was produced by cultivating the toxic strain *Aspergillus parasiticus* (NRRL 2999) in rice, according to a method developed by Shotweell *et al*. (1966), and was introduced into the formulation by replacing the carrier (sand). Aflatoxin levels in the experimental diets were quantified by HPLC using 1 μg/kg quantification level and recovery coefficient of 85.5%, performed at the LAPEMI laboratory.

**Table 1:** Nutritional and calculated composition of the basal diet for broilers

	Pre-starter	Starter	Grower	Finisher
Ingredient (g/kg)	1 to 7 days	8 to 21 days	22 to 38 days	39 to 45 days
Ground corn, 8%	576.20	584.20	644.70	671.20
Soybean meal, 46%	346.00	322.00	272.00	237.00
Meat meal, 43%	44.00	42.00	30.00	26.00
Soybean oil	7.50	25.00	31.50	43.00
Limestone, 38%	4.40	5.00	6.40	7.35
Ground salt	3.85	3.50	2.60	2.65
dl-methionine, 99%	3.10	3.00	2.50	2.10
l-lysine, 98%	2.05	2.30	2.15	2.20
l-threonine	1.10	1.20	0.85	0.70
Sodium bicarbonate	1.00	1.00	2.00	2.00
Vitamin/mineral supplement	5.00	5.00	5.00	5.00
Carrier	5.80	5.80	0.80	0.80
Calculated nutritional levels				
ME, MJ/kg	12.47	12.98	13.40	13.81
CP, g/kg	231.80	220.80	196.10	180.50
Ca, g/kg	10.50	10.10	9.00	8.06
aP, g/kg	5.20	5.00	4.20	3.90
Na, g/kg	2.20	2.10	1.90	1.90
SAA^6^, g/kg	10.20	9.80	8.70	7.90
Lysine, g/kg	14.00	13.50	11.80	10.80
Threonine, g/kg	9.50	9.20	8.00	7.30
Analyzed nutritional levels				
CP, g/kg	234.40	221.10	198.70	182.30
Fat, g/kg	44.60	54.00	61.70	67.90
Moisture, g/kg	109.50	108.40	112.60	114.80

ME = metabolizable energy; aP = available phosphorus; SAA = sulfur amino acids.

Mineral supplement per kilogram of diet: copper (100 g), iron (50 g), selenium (200 mg), zinc (50 g), manganese (70 g), iodine (1.2 g).

**Table 2:** Minimum levels of vitamins provided by the control vitamin supplement and OVN supplement for broilers

Vitamin	Unit	Pre-starter	Starter	Grower	Finisher
Control vitamin supplement					
Vitamin A	IU/ton feed	8 000 000	7 000000	6 000 000	5 000 000
Vitamin D_3_	IU/ton feed	2 400 000	2 200 000	2 000 000	1 000 000
Vitamin E	IU/ton feed	12 000	11 000	10 000	8000
Vitamin K_3_	mg/ton feed	2000	1600	1600	1600
Vitamin B_1_	mg/ton feed	2400	2000	1400	0
Vitamin B_2_	mg/ton feed	6000	5000	4000	2000
Vitamin _B6_	mg/ton feed	4000	3000	2000	0
Vitamin B_12_	mg/ton feed	14	12	10	5
Folic acid	mg/ton feed	1000	800	600	0
Niacin	mg/ton feed	40 000	35 000	30 000	20 000
Pantothenic acid	mg/ton feed	15 000	13 000	11 000	9000
Choline	g/ton feed	346	328	242	128
OVN supplement					
Vitamin A	IU/ton feed	13 000 000	11 250 000	11 250 000	11 250 000
Vitamin D_3_	IU/ton feed	4 000 000	4 000 000	4 000 000	4 000 000
Vitamin E	IU/ton feed	225 000	75 000	75 000	75 000
Vitamin K_3_	mg/ton feed	3500	3500	3500	3500
Vitamin B_1_	mg/ton feed	3500	2500	2500	2500
Vitamin B_2_	mg/ton feed	9000	8000	7000	7000
Vitamin B_6_	mg/ton feed	5000	5000	5000	5000
Vitamin B_12_	mg/ton feed	30	25	25	25
Folic acid	mg/ton feed	2250	2250	2250	2250
Niacin	mg/ton feed	70 000	70 000	65 000	65 000
Pantothenic acid	mg/ton feed	17 500	15 000	12 500	12 500
Choline	g/ton feed	550	550	575	575
Vitamin C	mg/ton feed	150 000	150 000	150 000	150 000
HyD^3^	mg/ton feed	69	69	69	69
Biotin	mg/ton feed	300	250	250	250

OVN = optimum vitamin nutrition; HyD = hydroxy vitamin D.

The levels of vitamins in premix are values provided by producer.

### Evaluated characteristics

Feed intake, weight gain and feed conversion were analyzed for birds aged 1 to 44 days. At day 45, two birds from each pen were slaughtered to determine the carcass characteristics: carcass yield, breast yield and leg (thigh + drumstick) yield. Breast samples were taken to determine meat quality (tenderness, water loss by dripping and lipid peroxidation), and the tibias was used to verify the incidence of BBS.

To determine tenderness, the breast muscles were placed in cooking parcels (Cryovac/CN-530) and cooked in a water-bath at 82°C. These were then stored for 24 h at 2°C. After cooling, the samples were cut hexagonally 2 × 1 × 1 cm, according to the method described by Froning and Uijtteenboogaart (1988). The samples with fibers positioned perpendicularly to the blades were cut with a texture meter (FTC Texture Test System model TP2) attached to a Warner Braztler with a speed of 20 cm/min and a cell load of 100 kg.

To determine water loss by dripping, the cuts were placed in polyethylene bags, which were labeled and sealed under atmospheric pressure and refrigerated for 48 h at 4°C. After this period, the cuts were weighed again (Bridi *et al*., 2003), and the second weight was expressed as a percentage of the initial weight.

Lipid peroxidation was determined based on the amount of malonaldehyde (**MDA**) using the method described by Tarladgis *et al*. (1960). The standard adopted was that of tetraethoxypropane 1.1′3.3″, which releases malonaldehyde during acid hydrolysis at a ratio of 1 mol : 1 mol. The results are expressed in MDA, defined as milligram of malonaldehyde per kilogram of sample.

The tibias were deboned and dried, preserving the periosteum. To determine the prevalence of BBS, bone lightness was analyzed by (L*) test. For this purpose, a Minolta 410R colorimeter was placed on the proximal epiphysis of the growth plate. The tibias received a score based on the light parameters, and their appearance was scored as acceptable (no darkening present in the bone – L* > 40), intermediate (presence of slight darkening in the bone – L* between 40 and 35) and unacceptable (severe darkening in the bone – L* < 35) (adapted from Baldo *et al*. 2013). The results are expressed as percentages of bones classified as acceptable, intermediate and unacceptable in each treatment.

### Statistical analysis

Data on performance, carcass yield, meat quality traits and BBS incidence were analyzed using the variance analysis method and the GLM process of the SAS (SAS Institute, Cary, NC, USA). In cases where the differences were significant, the means were compared using the Tukey test at 5% probability. For performance data, the statistical unit was the pen; and for meat quality, carcass yield and BBS incidence, the statistical unit was the animal.

## Results

There was an interaction for weight gain (*P* < 0.05) between vitamin levels and aflatoxin, showing that OVN levels in broiler diets helped to improve weight gain, compared with the control. In addition, the inclusion of a binder in the diet significantly improved feed conversion (Table 3).

**Table 3:** Broiler performance when fed different levels of vitamin in the diet with or without aflatoxin challenge

	Levels of vitamin supplementation								Probability							SEM
	Control				OVN				Vit	Afla	Bin	Vit × Afla8	Vit × Bin	Afla × Bin	Vit × Afla × Bin	
Afla, mg/kg	0	0	0.5	0.5	0	0	0.5	0.5								
Bin, mg/kg	0	10 000	0	10 000	0	10 000	0	10 000								
1 to 21 days																
FI, g	1159	1159	1153	1153	1161	1161	1155	1155	0.220	0.480	0.944	0.912	0.802	0.127	0.758	0.125
WG, g	804	803	801	799	809	808	806	804	0.005	0.002	0.388	0.004	0.775	0.291	0.137	0.430
FCR, g/g	1.44	1.44	1.44	1.44	1.44	1.44	1.44	1.44	0.943	0.943	0.859	0.836	0.785	0.822	0.921	0.050
1 to 44 days																
FI, g	5256	5242	5247	5234	5220	5206	5211	5199	0.312	0.420	0.878	0.521	0.664	0.234	0.278	0.125
WG, g	3159	3160	3144	3145	3182	3183	3167	3165	0.004	0.004	0.147	0.017	0.235	0.347	0.225	0.430
FCR, g/g	1.67	1.66	1.67	1.67	1.64	1.63	1.65	1.64	0.023	0.143	0.020	0.104	0.247	0.132	0.217	0.050

OVN = optimum vitamin nutrition; Vit = vitamin; Afla = aflatoxin; Bin = binder; FI = feed intake; WG = weight gain; FCR = feed conversion ratio.

Regarding carcass traits (Table 4), the treatment did not influence leg (thigh + drumstick) yield. However, OVN levels improved carcass yield compared with the control diet (*P* < 0.05). The presence of aflatoxin decreased carcass and breast yield (*P* < 0.05). There was a relationship between vitamin levels and the presence or absence of aflatoxin with carcass yield (*P* < 0.05). Broilers that were fed diets without aflatoxin had better carcass yield than diets with aflatoxin, and the supplementation of vitamins at higher than recommended (OVN) levels improved carcass yield in broilers that were fed diets with or without aflatoxin. The analysis of interactions between vitamin levels and the presence or absence of aflatoxins demonstrated that broilers that were fed a control diet with aflatoxin had lower carcass yield. Although the birds that were fed a diet with mycotoxin had lower carcass yield, the use of OVN improved carcass yield compared with the birds that were fed a control diet without aflatoxins.

**Table 4:** Broiler carcass characteristics, incidence of BBS and meat quality of broilers that were fed different levels of vitamin in the diets, with or without aflatoxin challenge, at 45 days of age

	Levels of vitamin supplementation								Probability							SEM
	Control				OVN				Vit	Afla	Bin	Vit × Afla	Vit × Bin	Afla × Bin	Vit × Afla × Bin	
Afla, mg/kg	0	0	0.5	0.5	0	0	0.5	0.5								
Bin, mg/kg	0	10 000	0	10 000	0	10 000	0	10 000								
Carcass traits, %																
Carcass	69.77	69.94	69.40	69.57	70.00	70.17	69.64	69.81	0.002	0.043	0.211	0.038	0.158	0.254	0.321	0.610
Breast	31.79	31.73	31.63	31.56	31.69	31.63	31.52	31.47	0.025	0.039	0.328	0.425	0.368	0.355	0.271	0.390
Legs	32.97	32.96	33.07	33.06	33.01	33.00	33.11	33.10	0.103	0.303	0.422	0.437	0.670	0.529	0.494	0.720
BBS, %																
Unacceptable (L*>40)	10.67	10.33	12.00	11.66	7.67	7.33	9.00	8.67	0.011	0.458	0.632	0.224	0.355	0.157	0.287	5.160
Intermediate (L*>35 to 40)	19.33	19.67	21.00	21.33	16.33	16.66	18.00	18.33	0.034	0.287	0.189	0.255	0.098	0.152	0.114	7.210
Acceptable (L*<35)	70.00	70.00	67.00	67.00	76.00	76.00	73.00	73.00	<0.001	0.047	0.690	0.871	0.129	0.224	0.199	11.04
Meat quality																
TBARS	2.83	2.85	2.95	2.97	1.49	1.51	1.61	1.63	0.003	0.018	0.348	0.388	0.412	0.255	0.221	3.360
Water loss, %	21.33	21.59	21.38	21.64	20.90	21.16	20.95	21.22	0.117	0.259	0.222	0.133	0.425	0.235	0.367	2.440
Tenderness, kgf	1.41	1.71	1.43	1.73	1.43	1.73	1.45	1.75	0.521	0.456	0.001	0.311	0.244	0.411	0.281	0.630

OVN = optimum vitamin nutrition; Vit = vitamin; Afla = aflatoxin; Bin = binder; BBS = black bone syndrome; TBARS = thiobarbituric acid reactive substances (measured as milligram of malonaldehyde per kilogram of sample).

L* indicates values of lightness.

OVN treatment decreased the frequency of unacceptable and intermediate levels of BBS (Table 4) and improved the percentage of acceptable bone lightness (*P* < 0.05). The analysis of breast quality showed that the treatments did not influence water loss by dripping (*P* > 0.05) (Table 4). The addition of aflatoxins in the diet increased MDA levels compared with a mycotoxin-free diet. OVN supplementation of the diet decreased the levels of MDA. Also, meat tenderness significantly decreased when binders were used in the diet. It is worth noting that the presence or absence of aflatoxins and the different vitamin levels had no effect on broiler breast tenderness.

## Discussion

The deficiency of one or more vitamins can cause metabolic disorders, leading to reduced productivity, slow growth and an increased incidence of diseases. Without vitamins, metabolic reactions slow and become ineffective (Mavromichalis *et al*., 1999). Increasing the levels of fat-soluble vitamins can boost immunity (Felix *et al*., 2009). The benefits of such supplementation were demonstrated in this experiment, since all diets with OVN significantly improved weight gain and feed conversion compared with the control diet, even when challenged by aflatoxin.

Although vitamin supplementation is known to be beneficial, some studies have removed the premix from the diet in the last week of the finishing stage to determine whether this cost-saving measure might affect poultry performance. Broilers that were fed diets without vitamin supplementation, from 28 to 49 days of age, showed decreased weight gain, feed efficiency and breast yield (Deyhim and Teeter, 1993). In addition, broilers that were fed vitamin-free premix diets 1 and 2 weeks prior to slaughter (42 days of age) had lower weight gain, although feed intake and feed conversion were not affected (Christmas *et al*., 1995). Also, Castaing *et al*. (2003) fed two levels of vitamin supplements to broilers and noted that the higher level (twice the commercial standard) led to a higher weight gain at 38 days (1919 g) compared to the lower level (1878 g). It is clear that adequate levels of vitamin supplementation must be provided to ensure good performance.

The performance of broilers that were fed diets with aflatoxin was lower than broilers that were fed mycotoxin-free diets. The literature describes several toxic effects caused by mycotoxins in birds, including poor performance, liver diseases, immunosuppression, hemorrhages, poor carcass quality and change in the relative weight of organs (Edds and Bordtell, 1983; Hygino da Cruz, 1996; Moreira, 2000). In addition, mycotoxins can reduce the humoral immune response, facilitating the growth of pathogens and hindering performance (Terrassi *et al*., 2005). Therefore, the use of additional vitamin supplementation was essential to minimize losses due to the presence of aflatoxin. According to Santúrio (2000), the biggest negative impact was seen when birds were fed aflatoxin during their first 21 days of life, and this impact on weight gain is irreversible until slaughter (42 days), results that were replicated in this study. According to Santúrio (2000), the relationship between aflatoxin and vitamins was not clear.

The addition of binders to the diets did not improve performance, but significantly decreased feed conversion. Binders reduced feed intake, but the weight gain of birds was maintained. Thus, when binders were used, mycotoxins had a reduced negative impact on performance. If mycotoxins are present in the diet, their impact on performance can currently only be mitigated by the use of toxin binders (Diaz *et al*., 2002; Oguz *et al*., 2002). The improvement in feed conversion observed in the present study may be explained by the fact of binders adhering to aflatoxin and blocking its absorption from the gastrointestinal tract, making it inert and non-toxic to animals (Batina *et al*., 2005).

This study showed a significant improvement in carcass yield when birds were fed OVN levels (70.31% OVN vitamin and 69.61% control vitamin levels) and aflatoxin-free diets (70.25% and 0 ppm; and 69.15% and 0.5 ppm). The percentage of breast yield was higher in birds that were fed aflatoxin-free diets (31.87% with 0 ppm and 31.38% with 0.5 ppm).

As expected, this study showed that aflatoxin contamination resulted in a drop in performance and carcass yield; however, higher levels of vitamins in the diet improved broiler performance and carcass yield even when birds were challenged by dietary aflatoxin.

The relative weights of the heart, liver, gizzard and pancreas were also analyzed in this study; however, the treatments had no impact on these characteristics (data not shown). This result contradicts the findings of Giacomini *et al*. (2006), who analyzed the relative weights of organs and carcasses and found that the size of hearts and livers of birds that were fed diets with aflatoxin increased; spleen and gizzard showed no significant difference in relative weight; and the weight of carcasses of birds that were fed diets with aflatoxin significantly reduced. The lack of impact on the relative weight of organs in this study might be explained by the dose of aflatoxin used. It is possible that the amount of aflatoxin in the diet was insufficient to lead to changes in the weights of the analysed viscera, and similar findings were reported by Ortatali *et al*. (2004), who intoxicated broilers for 42 days with 50 and 100 µg aflatoxin per kilogram of feed and found no difference in the relative weight of liver, kidneys, spleen, thymus and bursa of Fabricius. This was hypothesized to be due to a low aflatoxin dosage used.

Regarding the incidence of BBS, the number of acceptable ratings for lightness increased when the birds were fed OVN, whereas the presence of aflatoxin in the diet increased the number of intermediate readings. Aflatoxin can impact liver, kidneys and the organs involved in the biosynthesis of active forms of vitamin D, responsible for transferring calcium and phosphorus from the diet to the blood stream (Hamilton, 1984). Given the toxic effects of aflatoxin and, consequently, a decrease in the amount of active vitamin D, the level of calcium absorbed by the intestine and deposited in the bones decreases (Siloto *et al*., 2011). This can explain the effects of aflatoxin on bone lightness. In addition, the results show that OVN levels improved bone characteristics in broilers, decreasing the incidence of BBS.

Another important aspect relating to the benefits of feeding vitamins to birds is improved meat quality. As this study shows, the use of OVN in broiler feed decreased lipid peroxidation, a characteristic of much interest to the industry. The oxidation of fats, particularly of unsaturated fatty acids, is the main driver for poor meat quality. Regardless of the source, meats are susceptible to oxidative deterioration, which determines the shelf life of these products. However, several antioxidants, including ascorbic acid and alpha-tocopherol, protect lipids in the membranes from oxidation (Mordenti and Marchetti, 1996). Oxidation occurs in two stages: firstly, the oxidation of phospholipids and, secondly, the oxidation of triglycerides, which is directly related to the degree of unsaturation; the shelf life of meat depends on the oxidation of polyunsaturated fatty acids (Gandemer, 1997). Once lipid oxidation starts, several secondary reactions, such as the formation of free radicals, are triggered. In addition to off-flavor compounds determining the quality of meat, other reactions such as the formation of potentially toxic compounds, such as alcohols, ketones, peroxides and aldehydes, can hinder the safety and stability of meat, leading to nutrient loss and further promoting oxidative reactions (Gray *et al*., 1996; Nam *et al*., 1997). The adequate supplementation of antioxidant vitamins improves the stability of membrane structures. As a result, the meat is expected to be more stable. Providing broilers with high levels of natural antioxidants gives the poultry industry a simple method to improve stability against oxidation, sensory quality, shelf life and the acceptability of meats.

Finally, the use of OVN in broiler diets improves performance and carcass characteristics (yield and meat quality) even when aflatoxin is present in the diet, and it can effectively minimize the negative effects caused by mycotoxins in birds regardless of the addition of binders.

## Software and data repository resources

This article is part of a dissertation, which is deposited in an official repository with open access: http://www.teses.usp.br/teses/disponiveis/10/10135/tde-31012018-151337/pt-br.php
